# KLF5 promotes tumor proliferation and oxaliplatin resistance via chromatin remodeling in KRAS-mutated colorectal cancer

**DOI:** 10.20517/cdr.2025.110

**Published:** 2025-10-10

**Authors:** Zhuoqing Xu, Silei Sun, Han Gao, Runhua Feng, Xiaohui Shen

**Affiliations:** ^1^Department of General Surgery, Ruijin Hospital, Shanghai Jiaotong University School of Medicine, Shanghai 200025, China.; ^2^Institute of Medical Robotics, Ruijin Hospital, Shanghai Jiao Tong University School of Medicine, Shanghai 200025, China.; ^3^Shanghai Institute of Digestive Surgery, Shanghai 200025, China.; ^4^Department of Emergency, Ruijin Hospital, Shanghai Jiao Tong University School of Medicine, Shanghai 200025, China.; ^#^Authors contributed equally.

**Keywords:** Colorectal cancer, KRAS mutation, KLF5, oxaliplatin, chemotherapy resistance

## Abstract

**Aim:** Patients with KRAS-mutated colorectal cancer (CRC) frequently exhibit resistance to conventional chemotherapy and epidermal growth factor receptor (EGFR)-targeted therapies. This study investigates the role of the transcription factor KLF5 in mediating proliferation and chemoresistance in KRAS-mutated CRC, aiming to identify novel therapeutic strategies to improve treatment outcomes.

**Methods:** We analyzed the association between KLF5 expression, KRAS mutation status, and patient prognosis using CRC tissue microarrays and public datasets. Proliferative capacity and oxaliplatin sensitivity were compared between KRAS-mutated and wild-type patient-derived organoids. RNA sequencing and CUT&Tag sequencing were employed to assess KLF5-mediated chromatin accessibility and downstream transcriptional regulation in KRAS-mutated CRC cells. *In vitro* and *in vivo* functional studies were conducted using three pairs of KRAS-mutated CRC cell lines (with KLF5 knockdown or overexpression) to evaluate KLF5’s impact on proliferation, cell cycle progression, stemness, and oxaliplatin response.

**Results:** KRAS-mutated CRC demonstrated enhanced proliferative capacity and oxaliplatin resistance, accompanied by KLF5 upregulation. In KRAS-mutated CRC cells, KLF5 promoted chromatin accessibility to initiate downstream transcription programs regulating cell cycle progression, platinum drug resistance, and apoptosis. Mechanistically, KLF5 drives oxaliplatin resistance by promoting proliferation through upregulation of the CDK4/6-Cyclin D1 axis, enhancing stemness via LGR5 and Nanog, and activating the XIAP/Bcl-2-dependent anti-apoptotic signaling pathway. *In vivo* experiments further confirmed that KLF5-overexpressing KRAS-mutated CRC tumors exhibited accelerated growth and reduced oxaliplatin sensitivity.

**Conclusion:** This study reveals that aberrantly elevated KLF5 promotes proliferation and chemoresistance in KRAS-mutated CRC. Targeting KLF5 represents a promising strategy to enhance chemotherapeutic response in this aggressive CRC subtype, offering a rationale for clinical translation.

## INTRODUCTION

Colorectal cancer (CRC) is one of the most prevalent malignancies worldwide, characterized by sequential genetic alterations in genes such as *APC*, *RAS*, and *TP53*^[1]^. Approximately 40% of CRC patients harbor KRAS mutations^[2]^, leading to the constitutive activation of the KRAS protein and resistance to epidermal growth factor receptor (EGFR) inhibitors^[2]^. Current standard therapies for KRAS-mutated CRC include surgical resection, chemotherapy, and targeted agents. Oxaliplatin-based regimens (e.g., FOLFOX, combining oxaliplatin with 5-fluorouracil and leucovorin) remain first-line chemotherapy options^[3]^. However, chemoresistance is frequently observed in KRAS-mutated CRC, posing a significant clinical challenge.

KLF5, a zinc-finger transcription factor, plays pivotal roles in tumor cell proliferation, survival, apoptosis, and drug sensitivity^[4]^. Recognized as an oncogenic driver, KLF5 is frequently overexpressed in CRC and promotes tumor progression^[5]^. Our prior work demonstrated that the KLF5 inhibitor ML264 restores oxaliplatin sensitivity in chemoresistant CRC patient-derived organoids (PDOs) by suppressing the KLF5/Bcl-2/caspase3 anti-apoptotic axis^[6]^. Similar oncogenic and therapy-resistant roles of KLF5 have been reported in esophageal squamous cell carcinoma^[7]^, prostate cancer^[8]^, ovarian cancer^[9]^, and breast cancer^[10]^. Notably, KRAS-mutated CRC exhibits KLF5 upregulation, which correlates with aggressive tumor behavior^[11]^. These findings suggest that KLF5 may serve as a critical mediator of malignant progression and chemoresistance in KRAS-mutated CRC, highlighting its therapeutic potential.

Gene expression networks in cancer cells are orchestrated by master regulatory transcription factors (MRTFs), which govern hallmark oncogenic traits such as sustained proliferation, evasion of growth suppression, and resistance to cell death^[12]^. Emerging evidence positions KLF5 as an MRTF that regulates chromatin accessibility, epigenetic modifications, and transcriptional programs critical for tumor progression and microenvironment remodeling^[13,14]^. Thus, KLF5 may drive CRC progression and chemoresistance by reshaping chromatin landscapes and activating oncogenic transcriptional networks, offering a mechanistic basis for targeting KLF5 to improve therapeutic outcomes in KRAS-mutated CRC.

## METHODS

### Cell lines and culture

This study utilized KRAS wild-type (WT) (RKO, HT29) and KRAS mutant (SW1116, SW480, SW620, HCT116, DLD1, LOVO) CRC cell lines, all obtained from the American Type Culture Collection (ATCC) with STR authentication. Cells were cultured in RPMI-1640 medium supplemented with 10% fetal bovine serum (FBS) and maintained in a humidified incubator at 37 °C with 5% CO_2_, following ATCC-recommended protocols^[4]^. All cell-based experiments were regularly monitored for mycoplasma contamination.

### Patients and specimens

This study was approved by the Biomedical Ethics Committee of Ruijin Hospital, Shanghai Jiao Tong University School of Medicine, with written informed consent obtained from all enrolled CRC patients. Inclusion criteria required pathologically confirmed primary CRC without preoperative neoadjuvant therapy (radiotherapy or chemotherapy), and specimens were collected from laparoscopic surgical resections. Pathological TNM staging was performed according to the 2015 National Comprehensive Cancer Network (NCCN) guidelines.

### Establishment and culture of patient-derived organoids from CRC patients

Tissue processing and patient-derived organoids (PDOs) generation were performed as previously described^[6]^. Surgical specimens were washed with PBS containing penicillin-streptomycin, mechanically dissociated into small fragments, and digested with 0.5 mg/mL type IV collagenase in DMEM at 37 °C for 1 h. The digested suspension was sequentially filtered through 100 and 40 μm cell strainers, washed twice with PBS, and centrifuged to collect cells. Cell pellets were embedded in Matrigel and cultured with organoid medium containing 50 ng/mL EGF, 500 nM A83-01, 50 ng/mL Noggin, 3 μM SB202190, 10 nM prostaglandin E2, and 1 × B27. PDOs were passaged using TrypLE^TM^ Express to dissolve Matrigel.

### Drug sensitivity assay

Drug screening was performed using stably passaged CRC PDOs. Approximately 50 PDOs embedded in Matrigel were seeded into 96-well plates and exposed to oxaliplatin (0-100 μM, covering clinically relevant plasma concentrations) or dimethyl sulfoxide (DMSO) vehicle control. Bright-field images were captured at baseline and on days 1, 3, 5, and 7 to monitor growth dynamics. Cell viability was quantified using CellTiter-Glo® Luminescent Assay. Dose-response curves were generated by nonlinear regression modeling, with results expressed as half-maximal inhibitory concentration (IC_50_)^[15]^.

### PDO growth evaluation

PDO growth was assessed as previously described^[6]^. Bright-field images were captured on days 1, 3, 5, 7, 9, 11, and 13 post-Matrigel embedding. Orga0.noid viability was assessed using CellTiter-Glo® Lumi nescent Assay, where ATP-dependent luminescence (relative light units, RLU) quantitatively reflected proliferative activity. RLU trajectories across time points were analyzed to generate growth curves and calculate population doubling time.

### Histological staining analysis

Tissue specimens were fixed in 4% neutral buffered formalin, paraffin-embedded, and sectioned for H&E staining and immunohistochemistry (IHC). Antibodies used for IHC analysis included KLF5 and Ki-67. The TUNEL apoptosis detection kit was used according to the manufacturer’s instructions. IHC results were semiquantitatively scored by two independent pathologists using the H-scoring system under blinded conditions. The CRC tissue microarray used in this study, containing 75 paired tumor and adjacent normal tissue samples, was constructed and validated as described in our prior publication^[4]^.

### Bioinformatics analysis

The survival analysis was performed on the website https://kmplot.com/analysis/. The colon cancer database was used. RFS and OS were compared between KLF5-high and KLF5-low patients using the median expression level as the cut-off value.

### RNA extraction and quantitative real-time PCR (qRT-PCR)

Paired normal and tumor tissues from CRC patients were collected, flash-frozen in liquid nitrogen, mechanically dissected, and homogenized. Total RNA was extracted using TRIzol reagent (Invitrogen, Carlsbad, CA, USA) according to the manufacturer’s instructions. RNA concentration was quantified using a Nanodrop 2000 spectrophotometer (Thermo Scientific, USA). cDNA was synthesized from total RNA using HiScript III RT SuperMix (Vazyme), and quantitative PCR was performed using SYBR Green (Vazyme) in accordance with the manufacturer’s protocols. The qPCR primers were designed and supplied by Genewiz (China). The primer sequences were as follows: KLF5 Forward, 5’-ACACCAGACCGCAGCTCCA-3’; KLF5 Reverse, 5’-TCCATTGCTGCTGTCTGATTTGTAG-3’; GAPDH Forward, 5’-ACCACAGTCCATGCCATCAC-3’; GAPDH Reverse, 5’-TCCACCACCCTGTTGCTGTA-3’. GAPDH was used as the reference control gene. The relative mRNA expression levels were calculated using the 2-ΔCT method.

### Protein extraction and western blotting

Total proteins were extracted from CRC cells using radioimmunoprecipitation assay (RIPA) lysis buffer containing protease/phosphatase inhibitors and quantified by bicinchoninic acid (BCA) assay. Equal amounts of proteins were separated by sodium dodecyl sulfate-polyacrylamide gel electrophoresis (SDS-PAGE) and transferred onto polyvinylidene fluoride (PVDF) membranes. After 1 h blocking with 5% non-fat milk, membranes were incubated with primary antibodies against KLF5, CDK1/2/4/6, Cyclin D1/D2/E1, p21/p16, LGR5, Nanong, SOX2, XIAP, BCL2, and loading controls (GAPDH) at 4 °C overnight. Following TBST washes, HRP-conjugated goat anti-rabbit/mouse secondary antibodies were applied for 2 h at room temperature. Signals were developed with ECL substrate, captured by a Tanon imaging system, and quantified using ImageJ software with normalization to loading controls.

### siRNA transfection

KLF5-specific siRNA was transiently transfected into CRC cells using Lipofectamine^TM^3000 following the manufacturer’s protocol. Cells were harvested 48 h post-transfection, and knockdown efficiency was validated by qPCR assay. The most potent siRNA target sequence was selected to design the corresponding shRNA.

### Lentiviral vector construction and validation

KLF5 overexpression (LV5-EF1a-GFP/Puro-KLF5) and knockdown (LV3-pGLV-h1-GFP/puro-sh-KLF5) lentiviral vectors, along with control vectors (LV5-EF1a-GFP/Puro-Vector and LV3-pGLV-h1-GFP/puro-sh-NC), were constructed by Shanghai GenePharma Co. Ltd. (China). Western blotting confirmed KLF5 protein modulation efficiency.

### RNA-seq library preparation and bioinformatics analysis

Total RNA was isolated using TRIzol, treated with DNase I, and processed with Illumina Stranded mRNA Prep kit for library construction. Libraries passing Agilent 2100 Bioanalyzer QC were sequenced (150 bp paired-end) on Illumina NovaSeq 6000 (TIANGEN Biotech, China). Raw reads were quality-controlled via FastQC, trimmed using Trimmomatic, and aligned to GRCh38 with HISAT2. Gene expression quantification by featureCounts and differential analysis (|log_2_FC| ≥ 1, FDR ≤ 0.05) were performed using DESeq2 (v1.38.3). KEGG/GO enrichment of differentially expressed genes (DEGs) was analyzed via KOBAS (v3.0) and clusterProfiler (v4.6) with Bonferroni-corrected *P* ≤ 0.05^[12]^.

### CUT&Tag-Seq

The CUT&Tag assay was performed following established protocols^[12]^. Briefly, 10^5^ cells were gently washed twice with wash buffer and incubated with Concanavalin A-coated magnetic beads for 10 min at room temperature. After removing unbound supernatant, bead-bound cells were resuspended in dig wash buffer and incubated overnight at 4 °C with a 1:50 dilution of primary antibody (normal mouse IgG) or control IgG on a rotating platform. Following primary antibody removal, cells were incubated with a 1:100 dilution of Rabbit Anti-Mouse IgG for 60 min. After 2-3 washes in dig wash buffer, cells were treated with a 1:100 dilution of pA-Tn5 adapter complex in dig-med buffer (0.01% digitonin, 20 mM HEPES pH 7.5, 300 mM NaCl, 0.5 mM spermidine, protease inhibitor cocktail) for 1 h. Subsequent washes in dig-med buffer were followed by tagmentation in 10 mM MgCl2-containing buffer at 37 °C for 1 h. DNA was purified via phenol-chloroform-isoamyl alcohol extraction and ethanol precipitation. For library preparation, 21 μL of DNA was mixed with 2 μL of universal i5 and barcoded i7 primers, amplified using NEBNext HiFi 2× PCR Master Mix under the following conditions: 72 °C for 5 min (gap filling); 98 °C for 30 s; 14 cycles of 98 °C for 10 s and 63 °C for 30 s; final extension at 72 °C for 1 min. Libraries were purified with XP beads.

### Cell viability assay

Cell proliferation was assessed using CCK-8 assay: CRC cells were seeded in 96-well plates (2 × 10^3^ cells/well with 200 μL RPMI-1640 medium) and cultured for 24 h. Medium was replaced with fresh RPMI-1640 containing 10% CCK-8 reagent, followed by 2 h incubation at 37 °C in the dark. Absorbance was measured at 450 nm using a microplate reader. Relative cell viability was calculated against the 0 h baseline, with triplicate independent experiments.

### Colony formation assay

Cells were seeded in 6-well plates at 1 × 10^3^ cells/well and cultured in a 37 °C, 5% CO_2_ incubator for 14 days. After gentle PBS washing, colonies were fixed with 4% paraformaldehyde (room temperature, 30 min), stained with 0.5% crystal violet for 2 h, rinsed with deionized water, and air-dried. Colonies containing ≥ 50 cells were imaged using an inverted microscope and quantified with ImageJ software, with triplicate independent experiments.

### EdU cell proliferation assay

Cell proliferation was assessed using the EdU Cell Proliferation Kit. CRC cells were seeded in 6-well plates (5 × 10^4^ cells/well) and cultured in RPMI-1640 medium for 24 h before treatment. Both treated and control cells were incubated with 50 μM EdU working solution in complete medium at 37 °C for 2 h, protected from light. After fixation with 4% paraformaldehyde and permeabilization with 0.5% Triton X-100, cells were reacted with Alexa Fluor 555-conjugated azide for 30 min in the dark. Nuclei were counterstained with Hoechst 33342. Three random fields per well were imaged using a fluorescence microscope. The ratio of EdU-positive cells was quantified using ImageJ software, with triplicate independent experiments.

### Cell cycle analysis

Cells were fixed with 70% ice-cold ethanol, stained with 50 μg/mL propidium iodide (PI) and 100 μg/mL RNase A in the dark for 30 min. Analysis was performed on a BD FACSCanto II flow cytometer. DNA content histograms were analyzed to quantify the percentages of cells in the G0/G1 (diploid), S (DNA synthesis), and G2/M (tetraploid) phases, with triplicate experiments.

### Tumor spheroid formation assay

Cells were dissociated with 0.25% trypsin, resuspended in serum-free spheroid formation medium (DMEM/F12 supplemented with 20 ng/mL EGF, 20 ng/mL bFGF, and 2% B-27® Supplement), and filtered through 40 μm cell strainers to obtain single-cell suspensions. Cells were seeded at 500 cells/well in ultra-low attachment 96-well plates and cultured at 37 °C, 5% CO_2_ for 14 days with 50% medium replacement every 3 days. Spheroid morphology was observed using an inverted microscope, and spheroid formation rate was quantified using ImageJ software, with triplicate independent experiments.

### Flow cytometry analysis

CRC cells were digested into single-cell suspensions using 0.25% trypsin/EDTA. The cells were washed with staining buffer (PBS containing 5% FBS) and resuspended in flow tubes at a concentration of 1 × 10^6^ cells per 100 µL. Cell staining was performed using fluorescently conjugated primary antibodies for 30 min at 4 °C in the dark. The antibodies used targeted CD44, CD133, and CD166. Samples were analyzed using a flow cytometer (BD Biosciences), and data were processed with FlowJo software (Tree Star).

### Animal study

Four-week-old male BALB/c nude mice were obtained from Shanghai Laboratory Animal Center, CAS. Animal experiments were performed in strict compliance with the Guidelines for Animal Care and Use from Ruijin Hospital and Shanghai Resource Center of Laboratory Animals of the Chinese Academy of Sciences. All animal procedures in this study were performed at PHENOTEK Biotech (Shanghai) Co., Ltd, which is a commercially accredited animal facility commissioned by Ruijin Hospital. CRC cells (5 × 10^6^) were subcutaneously injected into the right flank. When tumor volume reached 50 mm^3^, mice were randomized into control (saline) and oxaliplatin groups (5 mg/kg) for intraperitoneal injection twice weekly over 3 weeks. Tumor dimensions were measured every 5 days using digital calipers. Terminal euthanasia by CO_2_ asphyxiation followed by cervical dislocation was performed, with excised tumors subjected to histopathological analysis (*n* = 3/group).

### Quantitative and statistical analysis

Data are presented as mean ± SD from three independent experiments. Flow cytometry data were analyzed using FlowJo V10.8.1. Statistical graphs were generated with GraphPad Prism 8.4.3. Two-group comparisons were performed using a two-tailed Student’s *t*-test or the Mann-Whitney *U* test (nonparametric data). Multiple groups were analyzed using one-way ANOVA or two-way ANOVA as the experimental design required. Statistical significance was defined as *P* < 0.05.

## RESULTS

### KRAS-mutated CRC exhibits enhanced proliferative capacity and oxaliplatin resistance, accompanied by KLF5 upregulation

**Figure 1:**
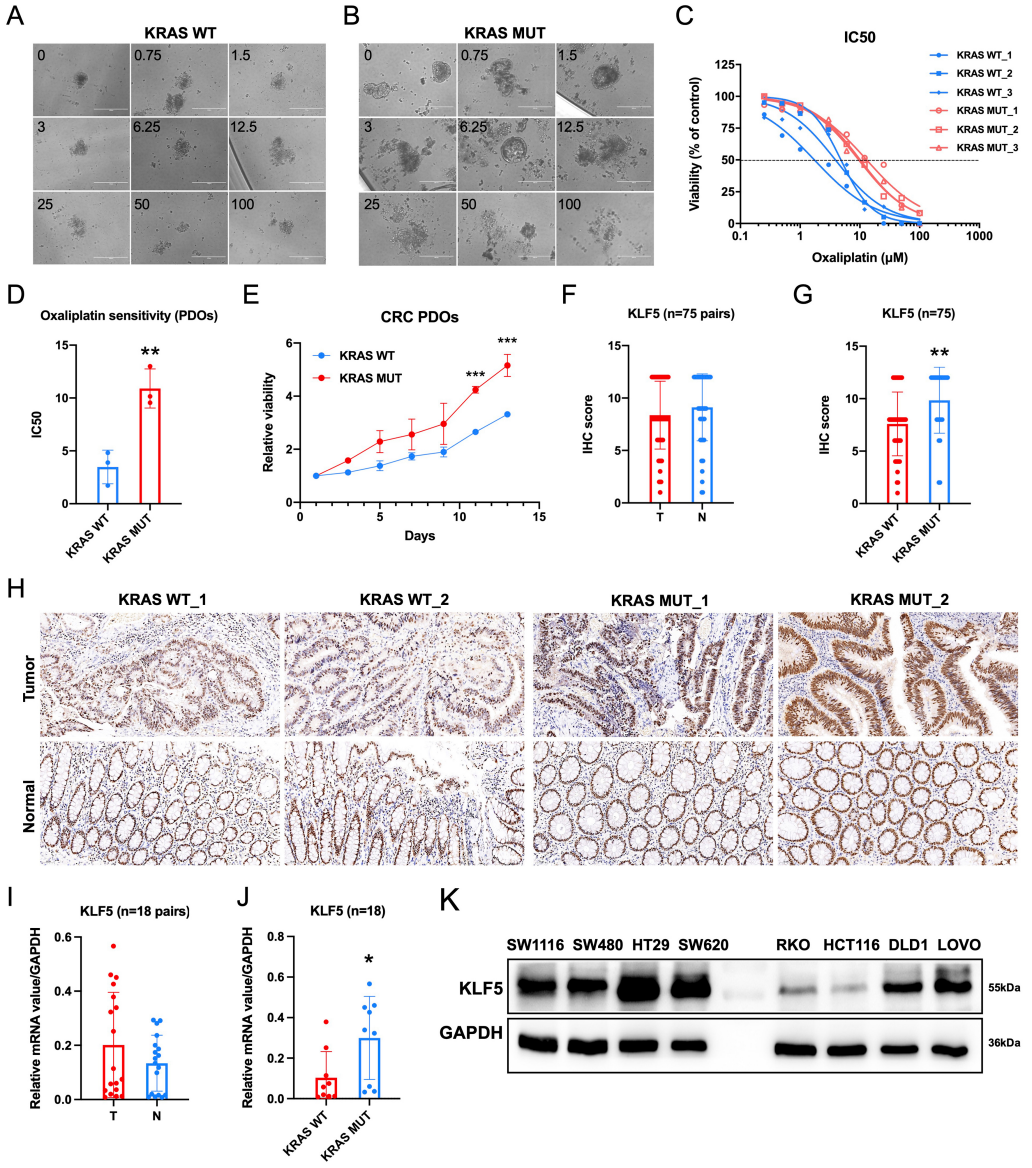
KRAS-mutant CRC exhibits elevated KLF5 expression, enhanced proliferation, and reduced sensitivity to oxaliplatin compared to KRAS wild-type CRC. (A) Oxaliplatin treatment in KRAS wild-type CRC; (B) Oxaliplatin treatment in KRAS-mutant CRC; (C and D) Dose-response curves demonstrating decreased oxaliplatin sensitivity in KRAS-mutant CRC, with significantly higher IC50 values compared to KRAS wild-type CRC; (E) Growth status of CRC PDOs, showing enhanced proliferative capacity in KRAS-mutant CRC PDOs; (F-H) Representative IHC staining images from a tissue microarray containing 75 paired CRC and adjacent N tissues. KLF5 expression shows no significant difference between T and adjacent N tissues, but is significantly upregulated in KRAS-mutant CRC; (I and J) qPCR analysis of KLF5 RNA expression levels in 18 paired CRC and adjacent N tissues. KLF5 mRNA expression shows no significant difference between T and adjacent N tissues, but is significantly upregulated in KRAS-mutant CRC; (K) KLF5 expression profiles in CRC cell lines. Data represent the mean ± SD, Student’s *t*-test. ^*^P < 0.05; ^**^P < 0.01; ^***^P < 0.001. CRC: Colorectal cancer; KLF5: Krüppel-like factor 5; IC50: half maximal inhibitory concentration; PDOs: patient-derived organoids; IHC: immunohistochemistry; qPCR: quantitative polymerase chain reaction; RNA: ribonucleic acid; mRNA: messenger RNA; SD: standard deviation; T: tumor; N: normal.

Patients with KRAS-mutated CRC exhibit a poor prognosis^[16]^. However, limited studies have systematically evaluated the predictive value of KRAS mutations in determining responses to conventional chemotherapy regimens^[17]^. We established PDOs from KRAS WT and mutant CRC tissues and evaluated their sensitivity to oxaliplatin. Drug response assays demonstrated that KRAS-mutant CRC PDOs exhibited significantly higher resistance to oxaliplatin compared to KRAS-WT counterparts, with elevated IC50 values [Figure 1A-D]. Furthermore, KRAS-mutant PDOs displayed enhanced proliferative activity [Figure 1E]. Our prior study^[6]^ revealed that ML264, a KLF5-specific inhibitor, restored oxaliplatin sensitivity in CRC PDOs by suppressing the KLF5/Bcl-2/caspase3 anti-apoptotic signaling pathway. To assess the clinical relevance of KLF5 in CRC, we evaluated both mRNA and protein expression levels of KLF5 in paired tumor and adjacent normal tissues [Figure 1F-J]. While no significant difference in KLF5 expression was observed between T and N tissues overall [Figure 1F, I], KRAS-mutant CRC tissues exhibited markedly higher KLF5 levels compared to KRAS-WT tumors [Figure 1G and J]. To address the clinical relevance of KLF5 expression, we performed survival analyses across multiple public databases [Supplementary Figure 1]. The results showed that in aggregated databases, KLF5 expression did not exhibit a significant correlation with overall survival (OS) or relapse-free survival (RFS). However, in certain specific datasets, higher KLF5 expression was associated with worse OS (GSE16446) [Supplementary Figure 1B] or RFS (GSE25066 and GSE12276) [Supplementary Figure 1F and G]. Collectively, these findings demonstrate that KRAS-mutated CRC is characterized by hyperproliferation, oxaliplatin resistance, and specific KLF5 upregulation, suggesting KLF5 as a potential mediator of these oncogenic traits in KRAS-driven CRC.

### KLF5 regulates cell cycle progression, platinum resistance, and apoptotic pathways in KRAS-mutated CRC

**Figure 2:**
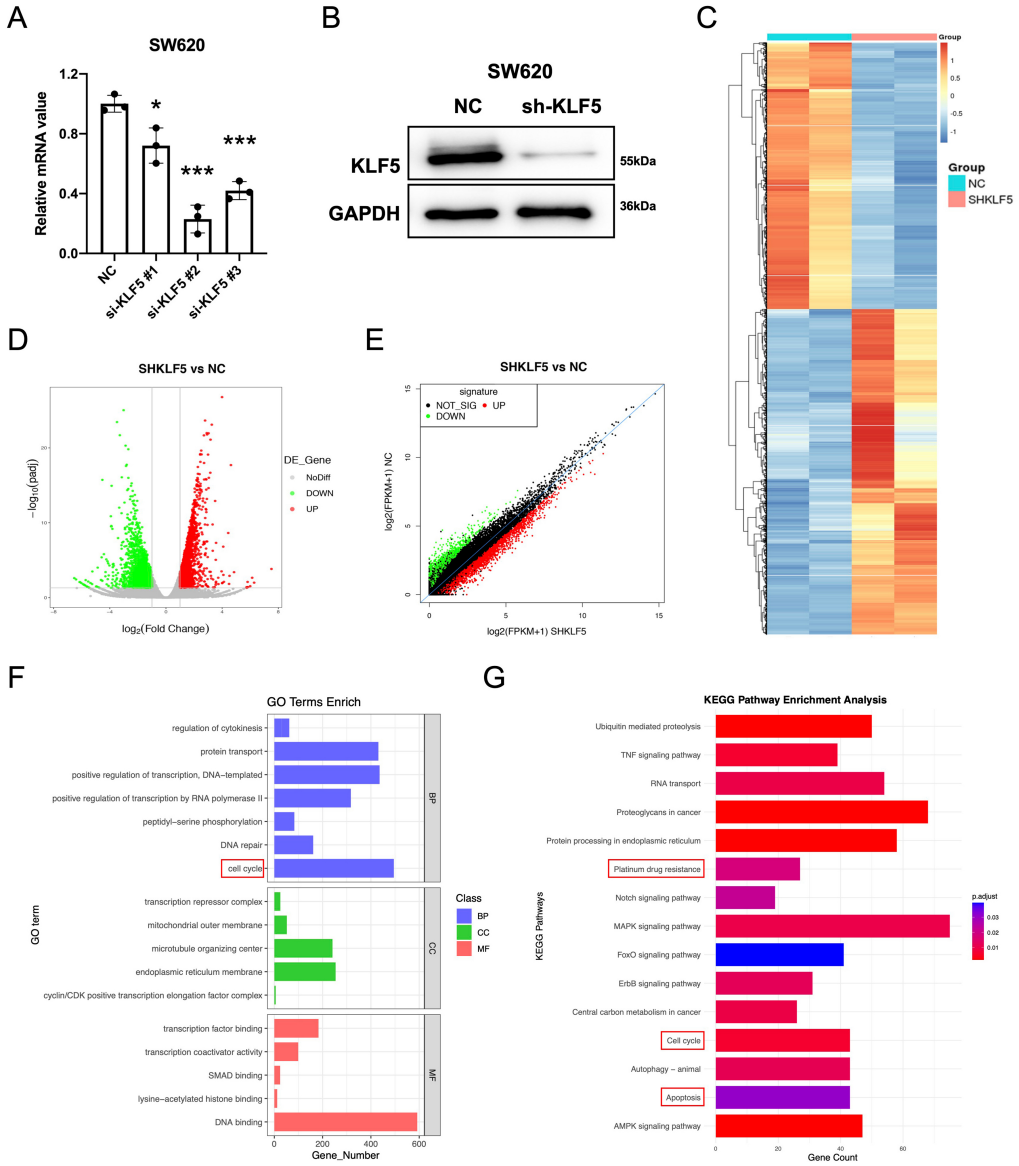
KLF5 regulates cell cycle, platinum drug resistance, and apoptosis-related pathways in KRAS-mutant CRC. (A) qPCR analysis of knockdown efficiency for different si-KLF5 sequences, with si-KLF5#2 showing the highest silencing efficacy; (B) Western blot validation of KLF5 knockdown efficiency; (C-E) RNA-seq analysis of differentially expressed genes (adjusted *P*-value < 0.05) in control (SHNC) and KLF5-knockdown (SHKLF5) KRAS-mutant CRC cells; (F) GO pathway analysis revealing top-ranked pathways altered upon KLF5 knockdown in SW620 cells; (G) KEGG pathway analysis highlighting the most significantly enriched pathways following KLF5 knockdown in SW620 cells. Data represent the mean ± SD, Student’s *t*-test. ^*^P < 0.05; ^**^P < 0.01; ^***^P < 0.001. KLF5: Krüppel-like factor 5; CRC: colorectal cancer; qPCR: quantitative polymerase chain reaction; siRNA: small interfering RNA; SHNC: short hairpin negative control; SHKLF5: short hairpin KLF5; RNA-seq: RNA sequencing; GO: Gene Ontology; KEGG: Kyoto Encyclopedia of Genes and Genomes; SD: standard deviation.

We first assessed KLF5 expression across eight CRC cell lines. In KRAS-WT cells, HT29 exhibited high KLF5 expression, while RKO showed low expression. Among KRAS-mutated cell lines, SW620, SW1116, HCT116, and LOVO displayed high KLF5 levels, whereas SW480 and DLD1 exhibited low expression [Figure 1K]. To investigate KLF5’s functional role, we performed lentivirus-mediated shRNA knockdown in SW620 (KRAS-mutant, highest KLF5 expression), achieving significant KLF5 suppression [Figure 2A and B]. RNA sequencing (RNA-seq) analysis of control (SHNC) and KLF5-knockdown (SHKLF5) cells identified DEGs [fold change (FC) > 1.5, FDR < 0.01; Figure 2C-E]. Gene Ontology (GO) enrichment analysis of DEGs revealed top-ranked pathways including cell cycle regulation, positive regulation of transcription, DNA repair, and cyclin/CDK-positive transcription elongation factor complex activity [Figure 2F]. Kyoto Encyclopedia of Genes and Genomes (KEGG) pathway analysis further highlighted significant enrichment in Cell cycle, MAPK signaling, Platinum drug resistance, and Apoptosis pathways [Figure 2G]. These findings collectively suggest that KLF5 may be involved in modulating critical oncogenic processes in KRAS-mutated CRC, including cell cycle progression, platinum-based chemoresistance, and regulation of apoptotic pathways.

### KLF5 enhances chromatin accessibility to activate downstream transcription in KRAS-mutated CRC

KLF5 is a transcription factor reported to be part of the core regulatory network in cancer, influencing chromatin accessibility, epigenetic modification, and gene expression patterns in tumor cells^[13]^. To further investigate the molecular mechanisms by which KLF5 regulates cell cycle progression, platinum-based chemoresistance, and apoptosis in KRAS-mutated CRC, we performed CUT&Tag sequencing in SW620 control (SHNC) and KLF5-knockdown (SHKLF5) cells to genome-widely profile KLF5 binding sites and their effects on chromatin accessibility. After stringent quality control, high-quality clean data were obtained, with total reads of 46,253,136 (SHNC) and 42,161,811 (SHKLF5). Alignment using the BWA tool revealed unique mapping rates of 26.37% (SHNC) and 16.03% (SHKLF5). Peaks were called with FDR < 0.05, identifying 18,086 and 10,305 enriched peaks in SHNC and SHNC groups, respectively. Genomic distribution analysis of peaks suggested KLF5’s potential functional targeting [Supplementary Figure 2A and B].

**Figure 3:**
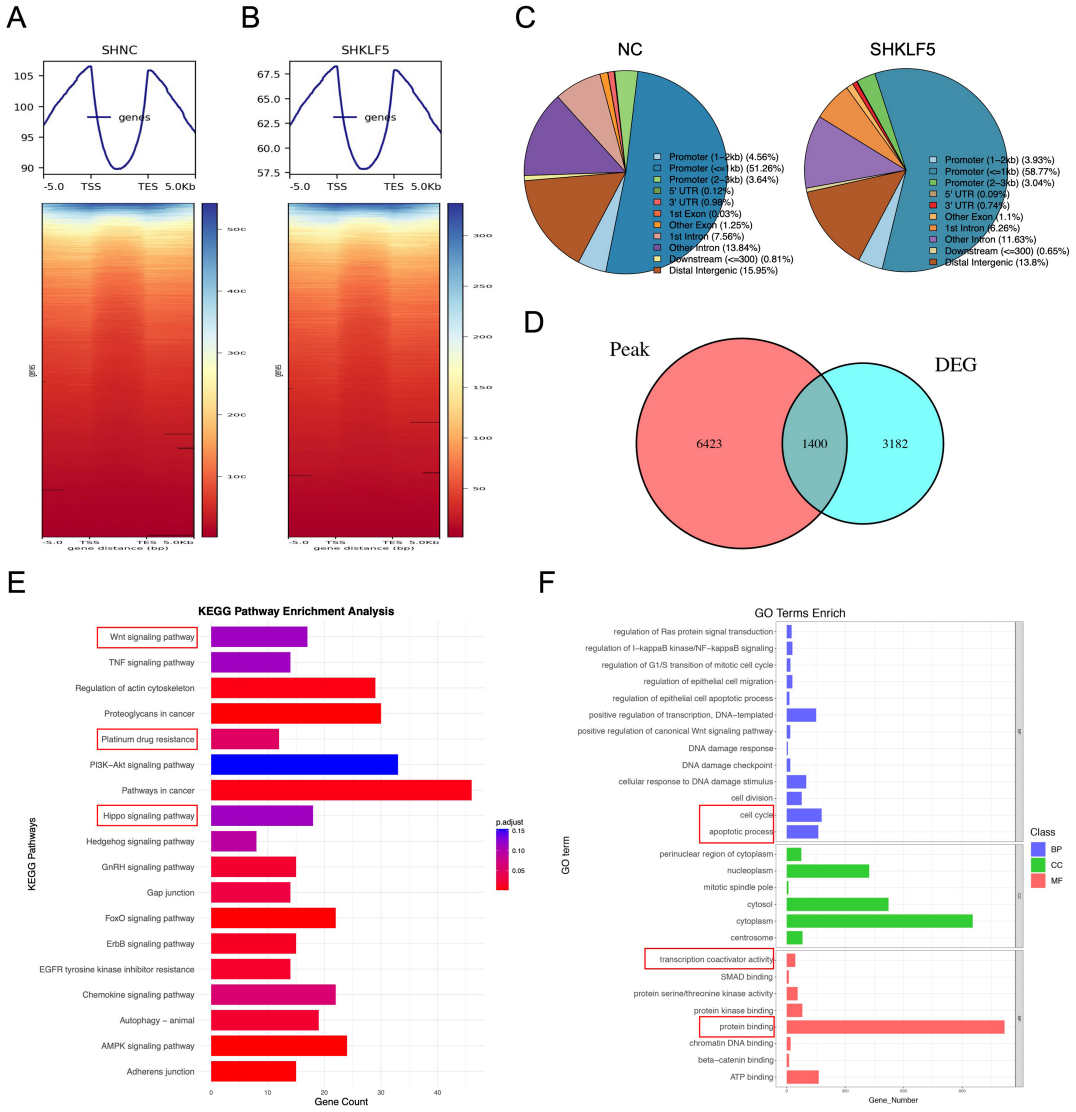
KLF5 promotes chromatin accessibility in KRAS-mutant CRC cells. (A and B) CUT&Tag sequencing analysis of control (SHNC) and KLF5-knockdown groups (SHKLF5). Signal distribution is calculated across TSSs, TESs, and flanking ± 3 kb regions, with corresponding heatmaps; (C) Pie chart summarizing the distribution of peaks across functional regions; (D) Venn diagram comparing differential peak-associated genes (SHKLF5 *vs*. SHNC) and RNA-seq-derived differentially expressed genes, identifying 1,400 overlapping genes; (E) GO pathway analysis of the overlapping genes; (F) KEGG pathway analysis of the overlapping genes. KLF5: Krüppel-like factor 5; CRC: colorectal cancer; CUT&Tag: cleavage under targets and tagmentation; SHNC: short hairpin negative control; SHKLF5: short hairpin KLF5; TSS: transcription start site; TES: transcription end site; GO: Gene Ontology; KEGG: Kyoto Encyclopedia of Genes and Genomes.

Using deeptools, we analyzed KLF5-associated chromatin signals across transcriptional start sites (TSSs), transcriptional end sites (TESs), and flanking 3 kb regions. Heatmaps demonstrated globally reduced signal intensity in the SHKLF5 group [Figures 3A and B, Supplementary Figure 2A and B], indicating KLF5’s role in promoting chromatin accessibility. Peak localization analysis revealed predominant enrichment of KLF5 at promoter regions in CRC cells [Figures 3C, Supplementary Figure 2C and D]. GO and KEGG pathway analyses of peak-associated genes highlighted key pathways linked to KLF5 activity [Supplementary Figure 2E-H].

Integration of differential peaks (SHNC *vs*. SHKLF5) with RNA-seq DEGs identified 1,400 overlapping genes [Figure 3D]. KEGG enrichment of these genes implicated KLF5 in regulating WNT signaling, Platinum drug resistance, and Hippo signaling [Figure 3E]. GO analysis further linked these genes to cell cycle regulation, apoptosis, transcription coactivator activity, and protein binding [Figure 3F]. Integrative Genomics Viewer (IGV) genome browser snapshots displayed prominent CUT&Tag peaks at the promoter regions of key target genes. Compared to the SHKLF5 group (KLF5 knockdown), the NC group (normal control) exhibited stronger peak signals [Supplementary Figure 3 A-D], indicating that KLF5 directly enhances chromatin accessibility at these target gene loci. Notably, aberrant activation of the WNT/β-catenin pathway drives tumor progression and therapy resistance^[18]^, while Hippo signaling modulates tumor growth and drug response through proliferation and apoptosis control^[19]^. These findings collectively demonstrate that KLF5 facilitates chromatin opening to initiate downstream transcriptional programs that mediate proliferation and oxaliplatin resistance in KRAS-mutated CRC.

### KLF5 promotes proliferation and cell cycle progression in KRAS-mutated CRC

**Figure 4:**
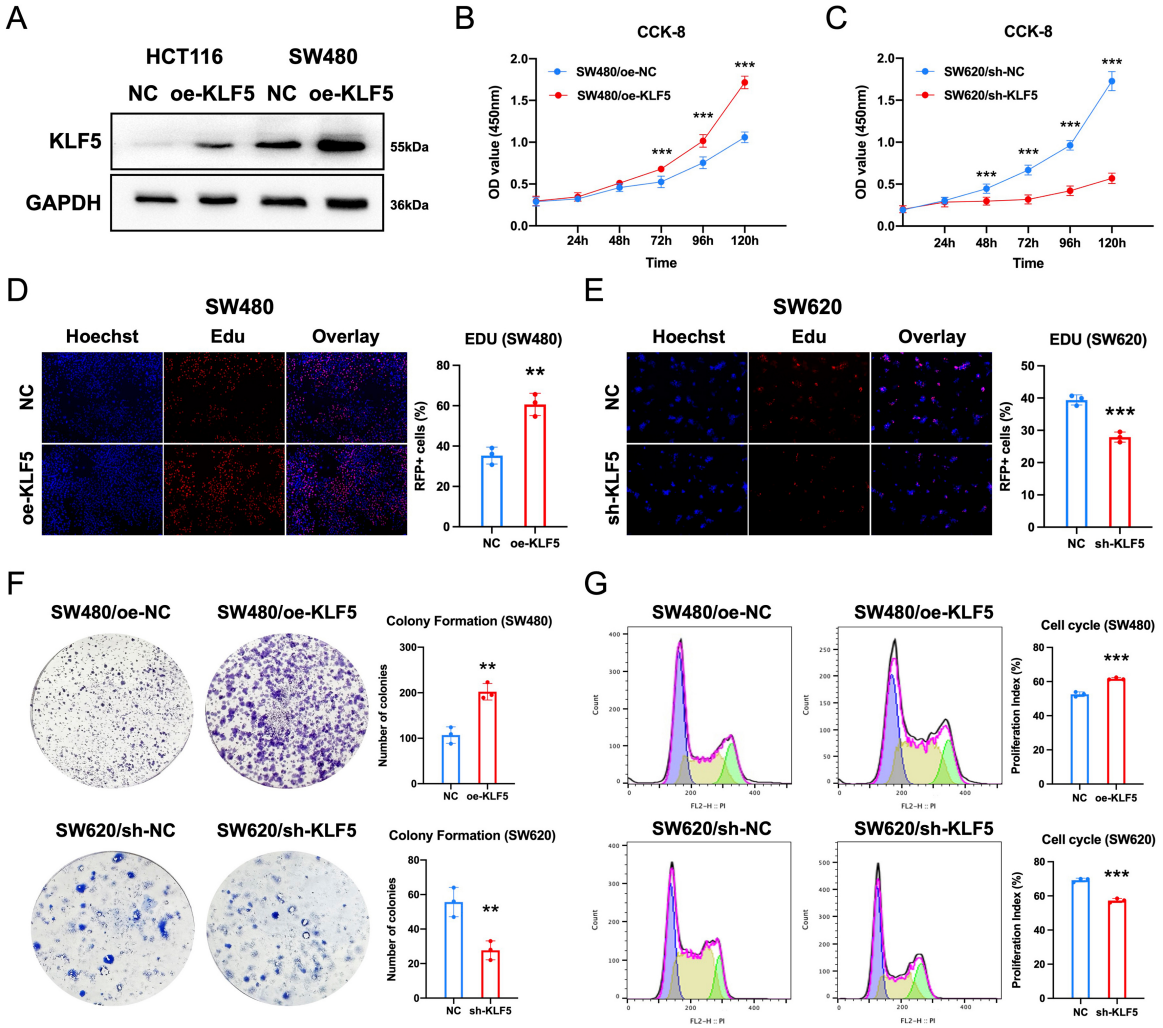
KLF5 promotes proliferation and cell cycle progression in KRAS-mutant CRC. (A) Western blot validation of KLF5 overexpression and knockdown efficiency; (B and C) CCK-8 assays assessing the effects of KLF5 on cellular proliferation; (D and E) EdU incorporation assays demonstrating KLF5-mediated regulation of proliferative activity; (F) Colony formation assay evaluating KLF5-dependent clonogenic capacity; (G) Cell cycle analysis revealing KLF5-driven modulation of cell cycle distribution. Data represent the mean ± SD, Student’s *t*-test. ^*^P < 0.05; ^**^P < 0.01; ^***^P < 0.001. KLF5: Krüppel-like factor 5; CRC: colorectal cancer; CCK-8: cell counting Kit-8; EdU: 5-ethynyl-2’-deoxyuridine; SD: standard deviation.

To functionally validate KLF5’s role in KRAS-mutated CRC, we transfected SW480 and HCT116 cells (KRAS-mutant, low KLF5 expression) with a KLF5 overexpression vector (oe-KLF5), achieving robust KLF5 upregulation as confirmed by Western blotting [Figure 4A]. Subsequently, we performed CCK-8 assays, EdU incorporation assays, and colony formation assays using stable CRC cell lines with KLF5 knockdown or overexpression, alongside their respective controls (SW480/oe-NC, SW480/oe-KLF5, SW620/sh-NC, SW620/sh-KLF5, HCT116/oe-NC, HCT116/oe-KLF5). CCK-8 proliferation assays demonstrated that KLF5 overexpression significantly enhanced cell viability, whereas KLF5 knockdown suppressed proliferation [Figure 4B and C, Supplementary Figure 4A]. Consistent results were observed in EdU incorporation assays [Figure 4D and E, Supplementary Figure 4B]. Colony formation assays further confirmed that KLF5 overexpression markedly increased clonogenic capacity, whereas KLF5 knockdown attenuated colony growth [Figure 4F]. Cell cycle analysis revealed that KLF5 overexpression accelerated cell cycle progression, whereas KLF5 knockdown induced G1-phase arrest [Figure 4G]. These data collectively demonstrate that KLF5 drives proliferation and cell cycle progression in KRAS-mutated CRC.

### KLF5 drives proliferation and cell cycle progression in KRAS-mutated CRC via upregulation of the CDK4/6-Cyclin D1 axis

**Figure 5:**
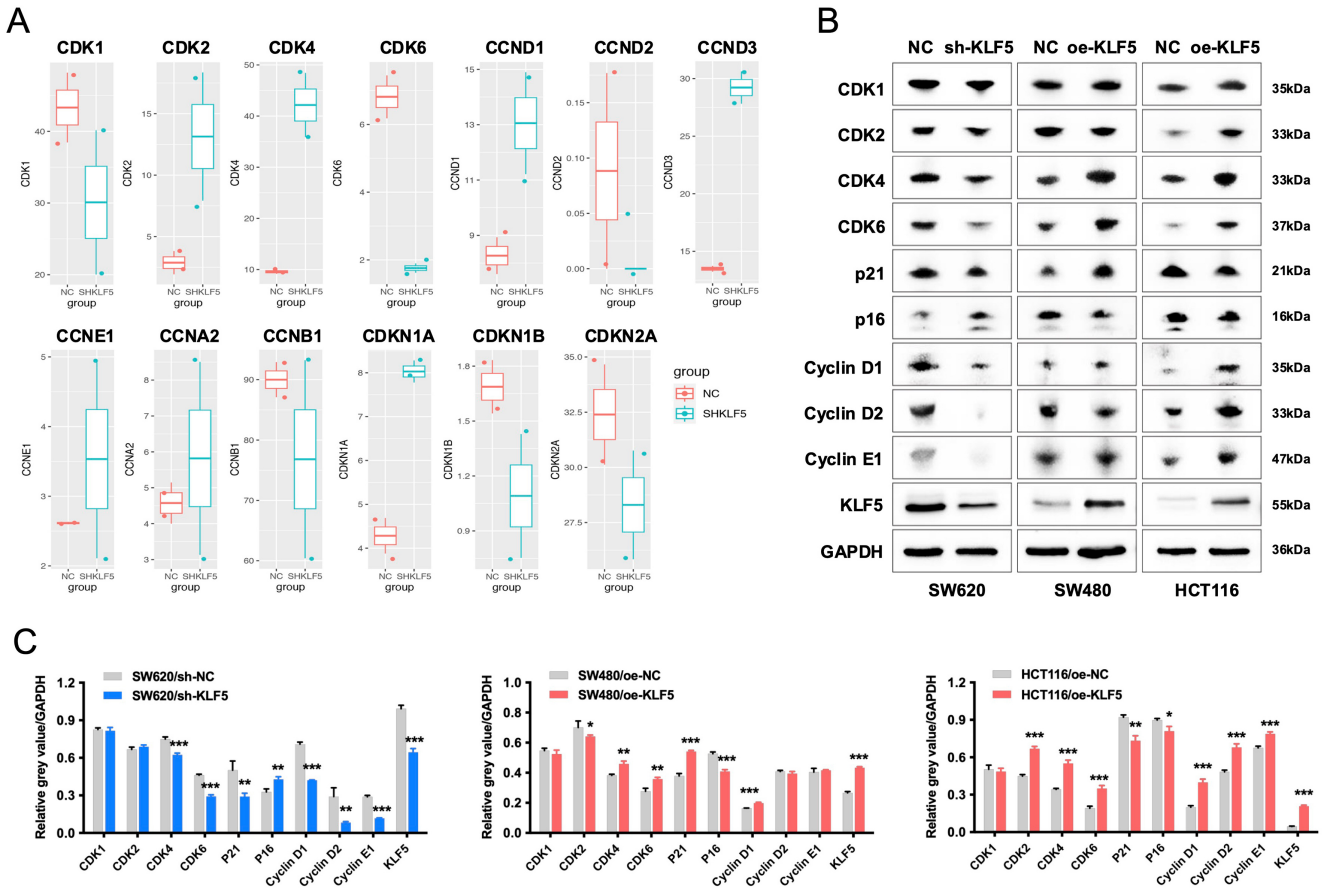
KLF5 drives proliferation and cell cycle progression in KRAS-mutated CRC via upregulation of the CDK4/6-Cyclin D1 axis. (A) RNA-seq analysis of differentially expressed cell cycle-related genes in KLF5-knockdown KRAS-mutant CRC cells; (B and C) Western blot analysis of KLF5, cell cycle regulators (CDK1, CDK2, CDK4, CDK6, Cyclin D1, Cyclin D2, Cyclin E1), and cell cycle inhibitors (p21, p16) in KRAS-mutant CRC cells, with GAPDH as a loading control. Data represent the mean ± SD, Student’s *t*-test. ^*^P < 0.05; ^**^P < 0.01; ^***^P < 0.001. KLF5: Krüppel-like factor 5; CRC: colorectal cancer; CDK: cyclin-dependent kinase; GAPDH: glyceraldehyde-3-phosphate dehydrogenase; SD: standard deviation.

Analysis of RNA-seq data from SW620 control (SHNC) and KLF5-knockdown (SHKLF5) cells revealed that KLF5 upregulates cell cycle-related genes (CDK1, CDK6, CCND2, CCNB1) [Figure 5A]. Western blotting further demonstrated that KLF5 overexpression in SW480 cells increased protein levels of CDK4, CDK6, Cyclin D1, and p21, whereas KLF5 knockdown in SW620 cells reduced the expression of these proteins. In TP53-wild-type, KRAS-mutant CRC cells (HCT116), KLF5 overexpression also upregulates CDK4, CDK6 and Cyclin D1, while downregulating the expression of cell cycle inhibitors p16 and p21 [Figure 5B and C]. CDK4 and CDK6, serine/threonine kinases, form complexes with Cyclin D1 to drive G1-to-S phase transition and promote proliferation^[20]^. These findings establish that KLF5 enhances KRAS-mutated CRC proliferation and cell cycle progression by activating the CDK4/6-Cyclin D1 axis.

### KLF5 promotes oxaliplatin resistance in KRAS-mutated CRC by enhancing tumor stemness and anti-apoptotic signaling

**Figure 6:**
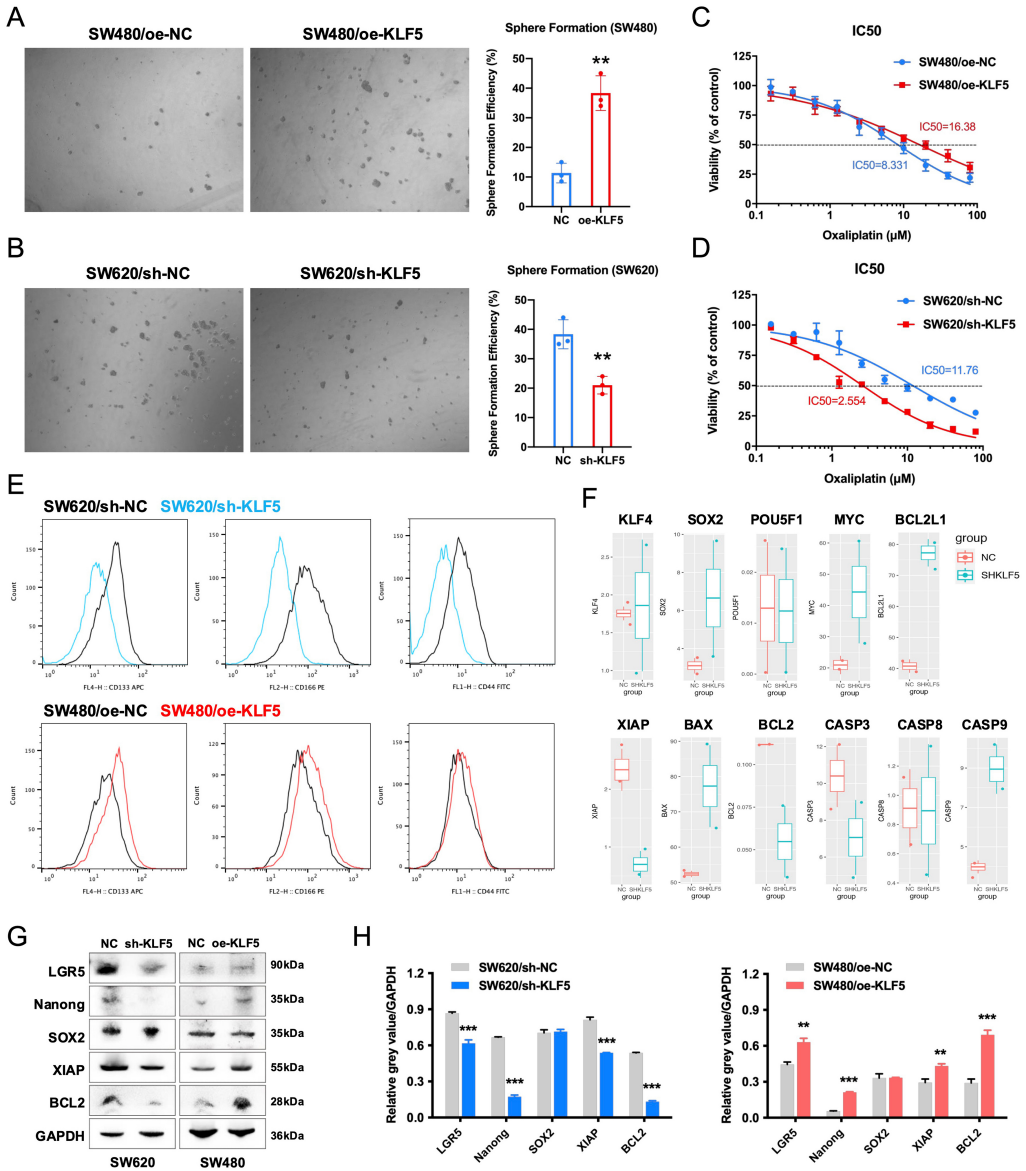
KLF5 enhances stemness and oxaliplatin resistance in KRAS-mutant CRC. (A and B) Sphere formation assays demonstrating KLF5-dependent regulation of cancer stem cell properties; (C and D) Dose-response curves of oxaliplatin treatment in KRAS-mutant CRC, highlighting KLF5-mediated drug resistance; (E) Flow cytometry analysis of stemness-related surface markers (CD133, CD166, and CD44) in CRC cells; (F) RNA-seq profiling of stemness- and apoptosis-associated genes altered by KLF5 knockdown in KRAS-mutant CRC cells; (G and H) Western blot analysis of stemness-related proteins (LGR5, Nanog, SOX2), and anti-apoptotic signaling molecules (XIAP and Bcl-2) in KRAS-mutant CRC cells, with GAPDH as a loading control. Data represent the mean ± SD, Student’s *t*-test. ^*^P < 0.05; ^**^P < 0.01; ^***^P < 0.001. KLF5: Krüppel-like factor 5; CRC: colorectal cancer; RNA-seq: RNA sequencing; LGR5: leucine-rich repeat-containing G-protein coupled receptor 5; SOX2: SRY-box transcription factor 2; XIAP: X-linked inhibitor of apoptosis protein; Bcl-2: B-cell lymphoma 2; GAPDH: glyceraldehyde-3-phosphate dehydrogenase; SD: standard deviation.

To investigate KLF5’s role in chemoresistance, we assessed cancer stemness using spheroid formation assays. KLF5 overexpression in SW480 cells significantly enhanced spheroid-forming capacity, while KLF5 knockdown in SW620 cells markedly suppressed this phenotype [Figure 6A and B]. Oxaliplatin sensitivity assays demonstrated that KLF5 overexpression reduced drug responsiveness in SW480 cells, whereas KLF5 knockdown sensitized SW620 cells to oxaliplatin [Figure 6C and D]. We then quantified the expression of stemness surface markers CD133, CD166, and CD44 using flow cytometry, demonstrating that KLF5 overexpression increased the expression of these stemness markers in SW480 cells, while KLF5 knockdown suppressed their expression in SW620 cells [Figure 6E].

Analysis of RNA-seq data from SW620 control (SHNC) and KLF5-knockdown (SHKLF5) cells revealed that KLF5 upregulates the expression of XIAP and BCL2 while suppressing the expression of BCL2L1, BAX, and CASP9 [Figure 6F]. Mechanistically, BCL2 maintains mitochondrial outer membrane integrity by inhibiting pro-apoptotic BAX activation, thereby preventing cytochrome c release^[21]^. XIAP directly binds caspases (e.g., CASP9, CASP3, CASP7) via its BIR domain to block apoptosis execution^[22]^. These anti-apoptotic processes have been demonstrated to mediate tumor resistance to oxaliplatin^[23-25]^. Our prior work demonstrated that KLF5-mediated upregulation of Bcl-2 suppresses caspase3-dependent apoptosis, contributing to oxaliplatin resistance in CRC^[6]^. Furthermore, Western blot analysis confirmed that KLF5 upregulates the expression of stemness-related genes (*LGR5* and *Nanog*) as well as anti-apoptotic signaling molecules (XIAP and Bcl-2) [Figure 6G and H]. These results demonstrate that KLF5 promotes oxaliplatin resistance in KRAS-mutated CRC by enhancing tumor stemness and activating anti-apoptotic signaling pathways.

### KLF5 promotes tumor growth and oxaliplatin resistance in KRAS-mutated CRC *in vivo*

**Figure 7:**
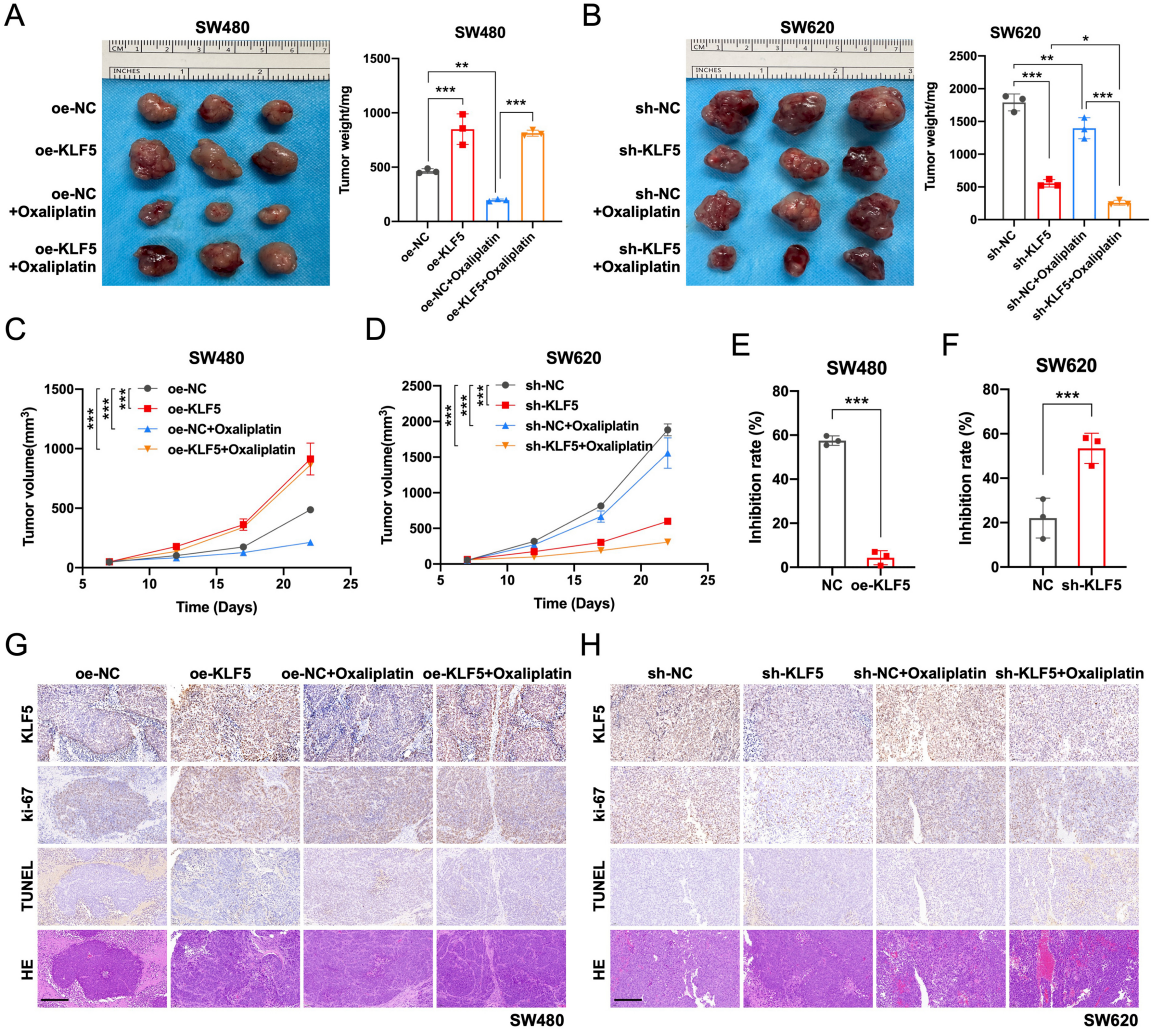
KLF5 drives tumor growth and suppresses oxaliplatin efficacy in KRAS-mutant CRC *in vivo*. (A and B) Xenograft tumor model established by injecting KRAS-mutant CRC cells into nude mice. Tumor volumes were calculated and plotted at the indicated time points (mean ± SEM); (C and D) KLF5 overexpression significantly promotes tumor growth and compromises oxaliplatin therapeutic effects in xenografts; (E and F) Assessment of Oxaliplatin tumor suppression efficiency in KRAS-mutated CRC tumors. KLF5 overexpression promoted Oxaliplatin resistance, while KLF5 knockdown synergistically enhanced tumor growth inhibition when combined with Oxaliplatin treatment; (G and H) IHC staining was performed to evaluate CRC tumor cell proliferation (Ki-67) and apoptosis levels (TUNEL). KLF5 overexpression significantly promoted CRC cell proliferation and suppressed oxaliplatin-induced apoptosis in CRC cells. Data represent the mean ± SD, Student’s *t*-test. ^*^P < 0.05; ^**^P < 0.01; ^***^P < 0.001. KLF5: Krüppel-like factor 5; CRC: colorectal cancer; SEM: standard error of the mean; IHC: immunohistochemistry; Ki-67: antigen Ki-67; TUNEL: terminal deoxynucleotidyl transferase dUTP nick end labeling; SD: standard deviation.

To validate KLF5’s role *in vivo*, we established subcutaneous xenograft models by injecting KRAS-mutated CRC cell lines (SW480/oe-NC, SW480/oe-KLF5, SW620/sh-NC, SW620/sh-KLF5) into the flanks of nude mice. When tumors reached 50 mm^3^, mice received intraperitoneal oxaliplatin (5 mg/kg, twice weekly). KLF5 overexpression in SW480 tumors significantly accelerated tumor growth and conferred oxaliplatin resistance, whereas KLF5 knockdown in SW620 tumors suppressed tumor progression [Figure 7A-D]. Notably, the combination of KLF5 knockdown and oxaliplatin treatment synergistically suppressed tumor growth, achieving maximal therapeutic efficacy [Figure 7E and F]. These findings confirm that KLF5 drives tumor progression and chemoresistance in KRAS-mutated CRC *in vivo*.

As shown by Ki-67 IHC staining, KLF5 overexpression in SW480 tumors significantly promoted CRC cell proliferation, while KLF5 knockdown in SW620 tumors inhibited CRC cell proliferation. TUNEL staining revealed that KLF5 overexpression in SW480 tumors suppressed Oxaliplatin-induced apoptosis of CRC cells, whereas KLF5 knockdown in SW620 tumors enhanced Oxaliplatin-induced apoptosis of CRC cells [Figure 7G and H]. These findings demonstrate that KLF5 promotes proliferation of KRAS-mutated CRC *in vivo* and mediates Oxaliplatin resistance through anti-apoptotic mechanisms.

## DISCUSSION

In the clinical management of KRAS-mutated CRC, frequent drug resistance is associated with aberrant sustained activation of signaling pathways such as EGFR^[26]^. Emerging evidence highlights the critical role of transcriptional regulatory networks in driving progression and therapy resistance in EGFR-mutated tumors^[27]^. Our study corroborates that KRAS-mutated CRC exhibits enhanced proliferative capacity and oxaliplatin resistance compared to KRAS wild-type counterparts. This underscores the potential of combining targeted therapies with conventional chemotherapy to improve therapeutic outcomes in KRAS-mutated CRC.

Our previous work demonstrated that ML264, a KLF5-specific inhibitor, restores oxaliplatin sensitivity in CRC PDOs by reactivating apoptotic responses via suppression of the KLF5/Bcl-2/caspase3 axis^[6]^. Notably, multiple KLF5 inhibitors - including those targeting triple-negative breast cancer^[28]^, CRC^[29]^, osteosarcoma^[30]^, and pancreatic cancer^[31]^ - have shown potent antitumor activity, suggesting broad therapeutic potential. Building on these findings, we identified elevated KLF5 expression in KRAS-mutated *vs*. wild-type CRC. This study systematically evaluates KLF5’s functional role in KRAS-mutated CRC, elucidates its mechanistic contributions to chemoresistance, and explores novel strategies to sensitize these tumors to chemotherapy.

Our study demonstrates that KLF5 promotes chromatin accessibility in KRAS-mutated CRC cells through RNA-seq and CUT&Tag sequencing analyses, initiating downstream transcriptional programs that regulate cell cycle progression, platinum drug resistance, and apoptotic pathways. Functional validation via *in vitro* and *in vivo* experiments further confirmed that KLF5 drives proliferation, cell cycle acceleration, stemness, and oxaliplatin resistance in KRAS-mutated CRC. Notably, we identified KLF5-mediated upregulation of the CDK4/6-Cyclin D1 axis as a critical mechanism for enhancing proliferation and cell cycle progression, while its suppression of apoptosis occurs through activation of anti-apoptotic proteins XIAP and BCL2. These findings align with prior studies highlighting KLF5’s regulatory roles in proliferation and apoptosis across cancers, yet they also underscore its context-dependent functional duality. For instance, KLF5 forms a complex with EHF/ELF3 in ovarian cancer to promote RAD51 transcription, enhancing homologous recombination repair and driving PARP inhibitor resistance^[9]^. In breast cancer, a KLF5-FOXO1-XPO1 positive feedback loop regulates cell cycle and proliferation in basal-like subtypes^[10]^.

Conversely, KLF5 has been reported as a potential tumor suppressor in certain epithelial cancers. In androgen receptor-positive prostate cancer models, KLF5 knockdown paradoxically enhances proliferation^[32]^, while in p53-mutant esophageal squamous cell carcinoma, KLF5 transactivates CDKN1A (p21) and NOTCH1, suppressing tumor growth^[33,34]^. These contrasting roles suggest that KLF5’s function is critically influenced by coexisting genetic alterations, particularly TP53 status. In our study, the KRAS-mutated CRC cell lines SW480 and SW620 harbor TP53 mutations. Although KLF5 upregulated p21 expression, no tumor-suppressive effects were observed, likely due to its dominant activation of the CDK4/6-Cyclin D1 axis, which overrides p21-mediated cell cycle arrest. This observation highlights the complexity of KLF5’s regulatory network, where its oncogenic or tumor-suppressive outcomes depend on tissue-specific signaling contexts and genetic backgrounds. Our findings thus provide mechanistic insights into KLF5-driven chemoresistance in KRAS-mutated CRC and underscore the therapeutic potential of targeting KLF5 or its downstream effectors to overcome platinum-based therapy resistance.

Although KLF5 has been implicated in CRC risk^[35]^ and is reportedly overexpressed in CRC tissues, correlating with disease progression and poor prognosis^[36]^, our cohort revealed no significant difference in KLF5 expression between tumor and adjacent normal tissues. Notably, however, KLF5 was specifically upregulated in KRAS-mutated CRC. Recent studies have identified KLF5 as a critical transcriptional regulator in this molecular subtype^[11]^. Furthermore, emerging evidence suggests that gene expression networks in cancer cells are governed by MRTFs, which orchestrate chromatin accessibility or cooperate with super-enhancers to sustain oncogenic programs such as proliferation, anti-apoptotic signaling, and drug resistance^[12]^. Through CUT&Tag sequencing, we demonstrated that KLF5 promotes chromatin remodeling in KRAS-mutated CRC cells, initiating transcriptional activation of pathways critical for proliferation and oxaliplatin resistance. Collectively, this work establishes KLF5 as a pivotal driver of malignant phenotypes in KRAS-mutated CRC.

Nevertheless, our study has several limitations. For instance, high KLF5 expression was observed in certain KRAS wild-type CRC cells (e.g., HT29, which carries a BRAF mutation), suggesting that its strong expression might be driven by aberrant signaling in the Ras/Raf pathway rather than by KRAS status itself. However, the upstream regulatory mechanisms controlling KLF5 expression were not sufficiently explored in this work. Future studies should delineate the interplay between KRAS mutations and KLF5 regulation, as well as clarify KLF5’s divergent roles in KRAS wild-type *vs*. mutant CRC. Additionally, validation of KLF5-targeted strategies in larger clinical cohorts and diverse preclinical models (e.g., patient-derived xenografts, organoids) is essential to confirm its therapeutic potential. Despite these limitations, our findings highlight the promise of KLF5 inhibition as a strategy to sensitize KRAS-mutated CRC to oxaliplatin, reinforcing KLF5’s candidacy as a therapeutic target in this aggressive subset of CRC.

## DECLARATIONS

### Availability of data and materials

The datasets supporting the conclusions of this article are included within the article and its Supplementary Materials. All data supporting our findings are available from the corresponding author upon reasonable request.

### Copyright

© The Author(s) 2025.
