# Supplementary material for: KLF5 promotes tumor proliferation and oxaliplatin resistance via chromatin remodeling in KRAS-mutated colorectal cancer
Source: Cancer Drug Resist. 2025 Oct 10;8:53. doi: 10.20517/cdr.2025.110 (PMC12539215; doi:10.20517/cdr.2025.110)
Supplement: Supplementary file 1 [file cdr-8-53-SupplementaryMaterials.pdf]

Supplementary Materials

# KLF5 promotes tumor proliferation and oxaliplatin resistance via chromatin remodeling in KRAS-mutated colorectal cancer

**Zhuoqing Xu[1,2,3,#], Silei Sun[3,4,#], Han Gao[1,3,#], Runhua Feng[1,3], Xiaohui Shen[1,3]**

[1]Department of General Surgery, Ruijin Hospital, Shanghai Jiaotong University School of Medicine, Shanghai 200025, China.
[2]Institute of Medical Robotics, Ruijin Hospital, Shanghai Jiao Tong University School of Medicine, Shanghai 200025, China.
[3]Shanghai Institute of Digestive Surgery, Shanghai 200025, China.
[4]Department of Emergency, Ruijin Hospital, Shanghai Jiao Tong University School of Medicine, Shanghai 200025, China.
[#]Authors contributed equally.

**Correspondence to:** Dr. Runhua Feng, Dr. Xiaohui Shen, Department of General Surgery, Ruijin Hospital, Shanghai Jiaotong University School of Medicine, Shanghai 200025, China. E-mail: fengrunhua@aliyun.com; xhshendoc@yeah.net

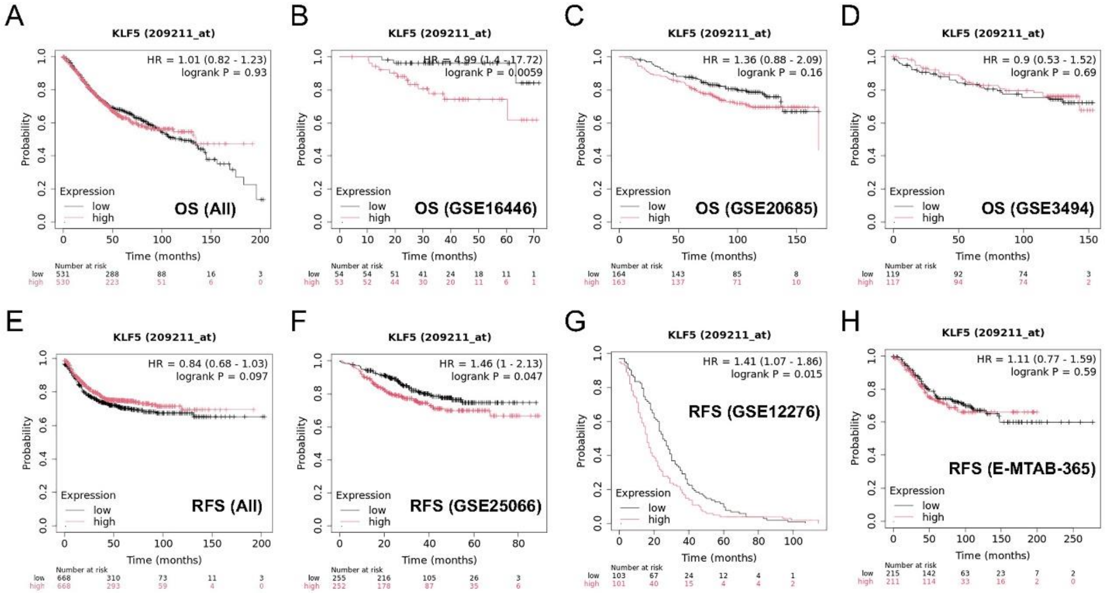


**Supplementary Figure 1.** Survival analysis of KLF5 expression in CRC patients. Analysis was performed using the KM-Plot online tool (https://kmplot.com/analysis/) with the CRC cohort. Patients were stratified into KLF5-high and KLF5-low groups based on the median expression level. (A-D) Comparison of OS between KLF5-high and KLF5-low groups. High KLF5 expression was associated with worse OS in dataset GSE16446; (E-H) Comparison of RFS between KLF5-high and KLF5-low groups. Elevated KLF5 expression correlated with reduced RFS in datasets GSE25066 and GSE12276. KLF5: Krüppel-like factor 5; CRC: colorectal cancer; KM-Plot: Kaplan-Meier Plotter; OS: overall survival; RFS: relapse-free survival.

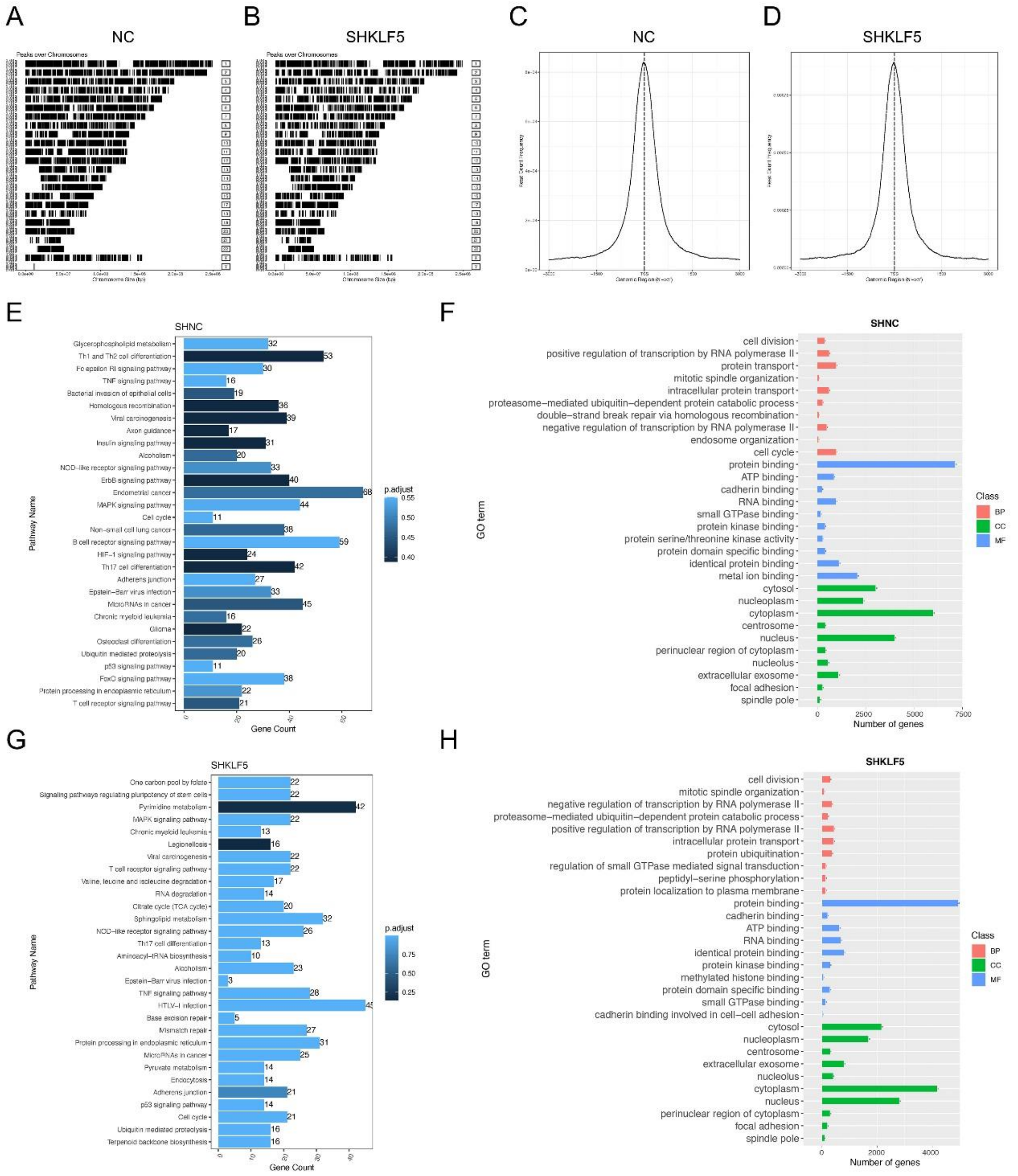


**Supplementary Figure 2.** KLF5 enhances chromatin accessibility in KRAS-mutant CRC cells. (A and B) Genome-wide distribution of detected peaks. The x-axis represents chromosomal positions (scaled by chromosome length, Mb), and the y-axis indicates the number of peaks identified at each genomic locus; (C and D) CUT&Tag sequencing of SW620 control (SHNC) and KLF5-knockdown (SHKLF5) groups, showing signal distribution relative to TSSs; (E and F) GO and KEGG pathway analyses of top-ranked pathways associated with peak regions in SW620 control (SHNC) cells; (G and H) GO and KEGG pathway analyses of top-ranked pathways linked to peak regions in KLF5-knockdown (SHKLF5) cells. KLF5: Krüppel-like factor 5; CRC: colorectal cancer; Mb: megabase; CUT&Tag: cleavage under targets

and tagmentation; SHNC: short hairpin negative control; SHKLF5: short hairpin KLF5; TSS: transcription start site; GO: Gene Ontology; KEGG: Kyoto Encyclopedia of Genes and Genomes.

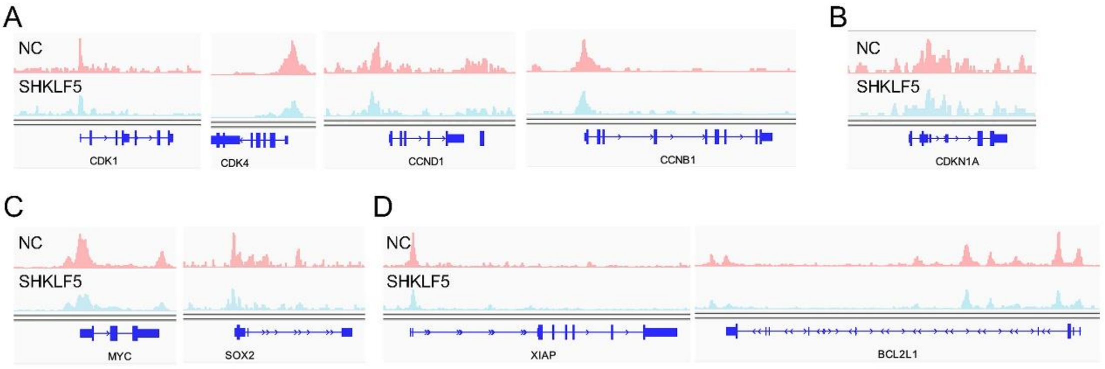


**Supplementary Figure 3.** KLF5 binding profiles at promoter regions of key target genes revealed by CUT&Tag sequencing. IGV genome browser snapshots of CUT&Tag signals in control (SHNC) and KLF5-knockdown (SHKLF5) SW620 cells at the promoters of proliferation-related (CDK1, CDK4, CCND1, CCNB1). (A) cell cycle inhibitory (CDKN1A); (B) stemness-associated (MYC, SOX2); (C) and anti-apoptotic (XIAP, BCL2L1); (D) genes. Stronger peak signals in the SHNC group compared to the SHKLF5 group indicate that KLF5 binding promotes chromatin accessibility at these loci. CUT&Tag data were visualized using the IGV. KLF5: Krüppel-like factor 5; CUT&Tag: Cleavage Under Targets and Tagmentation; SHNC: short hairpin negative control; SHKLF5: short hairpin KLF5; IGV: Integrative Genomics Viewer; CDK1: cyclin-dependent kinase 1; CDK4: cyclin-dependent kinase 4; CCND1: cyclin D1; CCNB1: cyclin B1; CDKN1A: cyclin-dependent kinase inhibitor 1A; MYC: MYC proto-oncogene; SOX2: SRY-box transcription factor 2; XIAP: X-linked inhibitor of apoptosis protein; BCL2L1: BCL2 like 1.

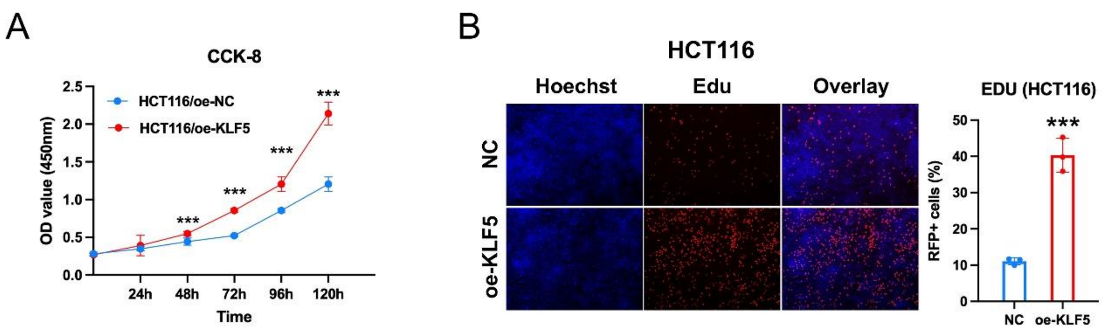


**Supplementary Figure 4.** KLF5 promotes proliferation in KRAS-mutant CRC. (A) CCK-8 assays assessing the effects of KLF5 on cellular proliferation; (B) EdU

incorporation assays demonstrating KLF5-mediated regulation of proliferative activity. Data represent the mean ± SD, Student's *t*-test. $^{***}P < 0.001$. KLF5: Krüppel-like factor 5; KRAS: Kirsten rat sarcoma viral oncogene homolog; CRC: colorectal cancer; CCK-8: Cell Counting Kit-8; EdU: 5-Ethynyl-2'-deoxyuridine; SD: standard deviation.
